# The fibronectin concentration that optimally maintains porcine satellite cells

**DOI:** 10.5713/ab.23.0108

**Published:** 2023-08-16

**Authors:** Jae Ho Han, Si Won Jang, Ye Rim Kim, Hoon Jang, Kwan Seob Shim, Hyun Woo Choi

**Affiliations:** 1 Department of Agricultural Convergence Technology, Jeonbuk National University, Jeonju 54896, Korea; 2 Department of Animal Science, Jeonbuk National University, Jeonju 54896, Korea; 3 Department of Life Science, Jeonbuk National University, Jeonju 54896, Korea; 4 Department of Animal Biotechnology, Jeonbuk National University, Jeonju 54896, Korea

**Keywords:** Fibronectin, Maintenance, Pax7, Porcine Satellite Cells, Proliferation

## Abstract

**Objective:**

‘Cultured meat’ has been suggested as means of solving the problems associated with overpopulation and gas emissions. Satellite cells are a major component in the production of cultured meat; however, these cells cannot be maintained *in vitro* over long periods. Fibronectin is a glycoprotein that affects biological processes such as cell adhesion, differentiation, and migration. Unfortunately, the characteristics of porcine satellite cells grown in a long-term culture when exposed to fibronectin-coated dishes are unknown. The objective of this study was to investigate the appropriate concentration of fibronectin coated dishes for proliferation and maintenance of porcine satellite cells at long-term culture.

**Methods:**

In this study, we isolated the satellite cells and fibroblast cells with pre-plating method. We next analyzed the cell doubling time, cell cycle, and rate of expressed paired box 7 (Pax7) and myogenic differentiation 1 (MyoD1) in porcine satellite cells cultured with 20 μg/mL of fibronectin-, gelatin-, and non-coated dishes at early and late passage. We then analyzed the proliferation of porcine satellite cells with various concentrations of mixed gelatin/fibronectin. We next determined the optimal concentration of fibronectin that would encourage proliferation and maintenance of porcine satellite cells in a long-term culture.

**Results:**

Doubling time was lowest when 20 μg/mL of fibronectin was used (as tested during an early and late passage). Levels of expressed Pax7 and MyoD1, assessed using immunocytochemistry, were highest in cells grown using fibronectin-coated dishes. The proliferation of gelatin/fibronectin mixed coatings had no significant effect on porcine satellite cells. The concentration of 5 μg/mL fibronectin coated dishes showed the lowest doubling time and maintained expression of Pax7.

**Conclusion:**

Fibronectin with 5μg/mL effectively maintains porcine satellite cells, a discovery that will be of interest to those developing the next generation of artificial meats.

## INTRODUCTION

Maintaining satellite cells *in vitro* is a critical component of the development of cultured meats. A number of countries have tried to solve the problems of environmental pollution and inadequate food supply, both of which stem from an increasing population [1]. Cultured meat (i.e., artificial meat) is a product intended to solve these problems [2]. Cultured meat requires a continuous supply of satellite cells. However, maintaining satellite cells *in vitro* remains challenging [3].

Satellite cells are muscle stem cells which can differentiate into muscle fiber. Satellite cells are located between the muscle fiber and basal lamina [4]. These satellite cells and muscle fibers are surrounded by blood vessels and an extra-cellular matrix (ECM), known collectively as the ‘niche’ or ‘micro-environment’. Within this niche, satellite cells are activated, and a signal governs their proliferation and differentiation into muscle fibers. As part of this process, collagen, laminin and fibronectin, part of the ECM, mediate cell-cell adhesion with integrin receptors and regulates activation of the signaling mechanism [5,6].

Researchers have attempted to develop techniques that effectively maintain and proliferate satellite cell *in vitro*. Growth factors within the satellite cells control proliferation and differentiation. Fibroblast growth factor (FGF) is known to be required for maintenance and regeneration of skeletal muscle [7]. β-Catenin, which is canonical of Wnt signals, interacts with myogenic differentiation (MyoD) and regulates differentiation [8]. It has also been reported that insulin like growth factor 1 (IGF-1) induces proliferation and muscle hypertrophy [9]. Within satellite cells, interleukin 6 is upregulated during muscle regeneration and promotes myogenic satellite cell proliferation [10]. Further studies have been conducted to examine cell signals within satellite cells. The mitogen-activated protein kinase (MAPK) pathway has been identified as the pathway by which myogenesis is initiated [11,12]. It has also been found that blocking the p38 MAPK signal pathway in bovine satellite cells enhances proliferation and paired box 7 (Pax7) expression and is essential to maintaining satellite cells and muscle regeneration [13].

ECM materials such as laminin, fibronectin, collagen are widely used *in vitro* on the coating dish in cell cultures to attach the cells and mimic the microenvironment in which cells interact [14]. Fibronectin is a glycoprotein that is produced in variety of cells, including fibroblasts, chondrocytes, myocytes, and synovial cells [15]. Fibronectin regulates cell behavior such as adhesion and migration by binding to integrin receptors [16]. It has been reported that different concentrations of ECM result in cells with divergent characteristics. A high density of collagen at the fibroblast increases the spreading capability, however, concentration above a certain level results in less cell spreading [17]. Osteoblast morphologies on the hydroxyapatite surface are also known to change depending on fibronectin concentration. Two types of oblates exist: end-on and an extended side-down configuration. Each has a respective size of 5,770 ng/cm^2^ and 168 ng/cm^2^. Each type is associated with morphological changes and adhesion capability. A low concentration of fibronectin on the hydroxyapatite attaches in a side-down configuration, while a high concentration of fibronectin on the hydroxyapatite attaches in an end-one configuration. As a result, MG63 cells on hydroxyapatite with a low concentration of fibronectin attach and spread better than when a high concentration of fibronectin is present [18]. However, the characteristics of porcine satellite cells grown through exposure to fibronectin in a long-term culture have remained unclear.

To determine the characteristics of porcine satellite cells grown on fibronectin-coated dishes, we compared short and long-term culture dishes with high-concentrations (20 μg/mL) of fibronectin, gelatin, and non-coated dishes for proliferation, cell cycle and immunocytochemistry (ICC). We also compared the proliferation of gelatin/fibronectin mixtures which had positive effect on chondorogenesis [19]. Next, we compared proliferation and maintenance when various concentrations of fibronectin were used with a long-term culture.

We ultimately found that satellite cells cultured on fibronectin-coated dishes showed greater proliferation and were better maintained. We then optimized the concentration of fibronectin for a long-term culture. We also observed no significant boost to proliferation to mixtures of gelatin and fibronectin. Fibronectin-coated satellite cells were effectively proliferated when 5 μg/mL and 20 μg/mL fibronectin were used during the long-term culture. We also showed that porcine satellite cells could be effectively maintained by expressing high levels of the *PAX7* gene in 1 μg/mL or 5 μg/mL of fibronectin in long-term culture. Surprisingly, 20 μg/mL of the fibronectin coated satellite cell exhibited the lowest Pax7 levels. In summary, 5 μg/mL was the most effective concentration at which fibronectin proliferated and was maintained.

Ensuring the sustainable culturing of satellite cells *in vitro* to produce cultured meat is essential. We found that the concentration of ECM changes the porcine satellite cells characteristics when grown in a long-term culture. Our result suggests that fibronectin at an appropriate concentration is a viable ECM on which to base future studies of satellite cells. Our study also provides an alternative method of maintaining porcine satellite cells.

## MATERIALS AND METHODS

### Animal care

All animal procedures were approved by the Animal Ethics Committee of Jeonbuk National University (JBNU,2020-0147). All experiments were performed in accordance with the ethical guidelines and regulations of Jeonbuk National University.

### Porcine satellite cell isolation

Muscle tissues were isolated from the front legs of a 1 day old porcine. 8 muscle tissues were cut off to 1 g and minced with surgical scissors. Tissues were then dissociated with Digest Solution containing Dulbecco’s modified eagle medium (DMEM)/F12 (#11320-033; Gibco, Carlsbad, CA, USA), 0.25% Trypsin-EDTA (TE) (#25200-072; Gibco, USA), 10% penicillin-streptomycin (PS) (#15140-122L; Gibco, USA), Collagenase D (#11088858001, 2 mg/mL; Roche, Indianapolis, IN, USA), and DispaseII (#4942078001, 1 U/mL; Roche, USA) for 30 min at 37°C. The mixture was titrated every 5 min. After dissociation the muscle cells were filtered through a 100 μm strainer followed by a 70 μm cell strainer. The cells were neutralized by Neutralized media containing DMEM (#11885092; Gibco, USA), 15% fetal bovine serum (FBS) (16000-044), and 1% PS. Cells were centrifuged at 1,500 rpm for 5 min at 4°C. The pellet was then reconstituted with ACK lysing buffer (#A10492-01; Gibco, USA) by removing red blood cells and incubating in ice for 5 min. Then cells were again centrifuged at 1,500 rpm for 5 min at 4°C. Cells were then reconstituted with culture media containing (DMEM/F12, 15% FBS 1% penicillin-streptomycin-glutamine (PSG) (#10378016; Gibco, USA) and basic fibroblast growth factor (bFGF) (#13256-029, 10 ng/mL; Gibco, USA). The porcine satellite cells were separated using the pre-plate method. Cells were seeded in a 100 mm dish coated with 0.1% gelatin (G1319; Sigma-Aldrich, St. Louis, MO, USA) and incubated at 37°C in a 5% CO_2_ incubator for 1 hour. After 1 hour, the medium (suspension cell) was collected and transferred to a new 100 mm dish coated with 0.1% gelatin.

### Porcine satellite cell culture

Cells were coated with 0.1% gelatin and various concentrations of fibronectin (#FC010; Sigma-Aldrich, USA). Cells were seeded in 60 mm and 35 mm dishes, with 4 wells and 96 wells, in 5×10^5^, 1.8×10^5^, 5×10^4^, and 5×10^3^ configurations. Media was changed every day with culture media and passaged every 3 days.

### Doubling time analysis

Porcine satellite cells were seeded in 35 mm dishes with 0.1% gelatin, various concentrations of fibronectin (1, 5, 20 μg/mL) and non-coated. Culture media was changed every day and cells were counted after 3 days of culture. After 3 days, cells were detached by 0.25% TE and each sample was counted using an inverted microscope with a hemocytometer. The experiment was performed in triplicate. The time taken to double three times were measured using a doubling time calculation:


$$\text{Doubling time}=\text{Duration }(\text{h})\times \log_{2}(\frac{final\;number\;of\;cell}{initial\;number\;of\;cell})$$

### Cell growth analysis

Porcine satellite cells were seeded in a 96 well with various concentrations of gelatin/fibronectin mixes. Porcine satellite cells were seeded in every well at a density of 5×10^3^ cells. Cells were cultured in culture media for 3 days. Growth was identified using a cell counting Kit-8 (CCK-8) (#CK04-11; Dojindo, Kumamoto, Japan). Cells were treated with CCK-8 solution consistent with the manufacture instructions and incubated at 37°C for 3 hours. Growth was measured using a microplate reader with a wavelength of 450 nm.

### Cell cycle analysis

Gelatin, fibronectin, and various concentrations of gelatin/fibronectin mix were collected at passage 2 (early) and passage 7 (late). Porcine satellite cells were seeded in a 35 mm dish with a density of 18×10^4^. For cell cycle analysis, passage 2 cells and passage 7 cells were detached using 0.25 TE and neutralized with neutralize media. Cells were then washed with cold PBS (containing 1% bovine serum albumin [BSA]) and fixed with 70% ethyl alcohol for 5 min at 4°C. Cells were centrifuged 850×g at 4°C for 5 min. Ethanol was removed and twice washed with PBS. After washing, 100 μg/mL of RNase A (#70856; Sigma-Aldrich, USA) was added, after which 25 μg/mL of propiodium iodide (PI) (#421301; Bio Legend, San Diego, CA, USA) was added with PBS. Cells were then analyzed with an FACS Calibur (Becton Dickson, Franklin, NJ, USA) with a blue laser (excitation 488 nm) installed at the Center for University Research Facility (CURF) at Jeonbuk National University.

### Immunofluorescence staining and the ratio of cultured porcine satellite cells

Gelatin, fibronectin, and non-coated Passage 2 and Passage 7 cells were seeded in a 4 well plate with a density of 5×10^4^ and cultured for three days. After three days, cells were twice washed in PBS. Cells were fixed overnight by 4% paraformaldehyde at 4°C. After fixation cells were washed with PBS three times, then incubated in blocking solution containing Washing solution + 3% BSA (Bovogen, Keilor East, Australia, Bovostar) and washing solution, 0.3% Triton X-100 (10010-023; Gibco, USA), as well as PBS, for 2 hour. After blocking, cells were washed with Washing solution. Cells were then stained overnight with primary antibodies against anti-MyoD1 (polyclonal, 1:200; Proteintech, Rosement, IL, USA) and anti-Pax7 (Pax7 monoclonal, 1:50; DHSB, Iowa, IA, USA) at 4°C. Cells were incubated with Alexa488 anti-mouse (Molecular Probes; Eugene, OR, USA) antibodies and Alexa586 anti-rabbit (Molecular Probe, USA) antibodies at room temperature for 2 hours. 4′6-diamidino-2-phenylindole (DAPI) with 1 μg/mL was stained for 10 min. After DAPI staining, cells were washed with washing solution for 10 min. The analysis was performed with a Leica 9900, with images captured in triplicate. Three students counted green-, red-, and blue-fluorescent cells from the same captured images. The outcomes are presented as number of Pax7 fluorescence/number of DAPI fluorescence, and number of MyoD1 fluorescence/DAPI fluorescence.

### Gene expression analysis by quantitative real-time polymerase chain reaction

RNA was extracted from cells using an AccuPrep Universal RNA Extraction kit (Bioneer, Seoul, Korea). The 1 μg of total RNA was reverse transcribed with an Accupower CycleScript RT Premix (Bioneer, Korea). Relative gene expression was performed in triplicate using Powerup SYBR Green Master Mix (Applied Biosystems, Waltham, MA, USA). The primers used for quantitative real-time polymerase chain reaction (qRT-PCR) were GapDH sense 5′-ACCCAGAAGA CTGTGGATGG-3′, GapDH antisense 5′-AAGCAGGGAT GATGTTCTGG-3′, Pax7 sense 5′-TCCAGCTACTCCGA CAGCTT-3′, Pax7 antisense 5′-TGCTCAGAATGCTCAT CACC-3′, MyoD1 sense 5′-GTGCAAACGCAAGACCAC TA-3′, and MyoD1 antisense 5′-GCTGATTCGGGTTGC TAGAC-3′.

### Statistical analysis

All experiments were performed three times and all data was collated and expressed as means±standard error of the mean. Statistical tests were conducted using SAS software version 9.4 (SAS Institute Inc., Cary, NC, USA), and tests for statistical differences were performed with Student’s *t*-test or analysis of variance, followed by Duncan’s multiple range test for post-hoc comparisons. A p-value<0.05 was regarded as significant.

## RESULTS

### Porcine satellite cells grown on fibronectin-coated dishes were effectively proliferated *in vitro*

**Figure 1:**
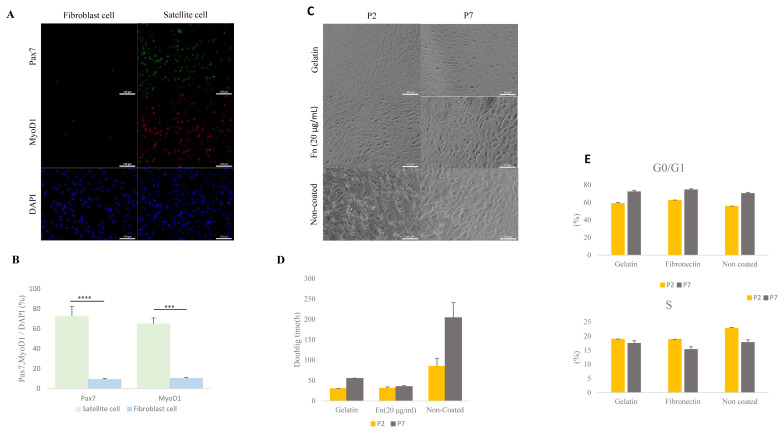
Comparative analysis of isolated porcine satellite cells form muscle tissue and comparative analysis of cell morphology, doubling time, and cell cycle with gelatin-, 20 μg/mL fibronectin (fn)– and non-coated satellite cells. (A) Isolation of porcine satellite cell using pre-plating. Morphology and immunocytochemistry of satellite cells and fibroblast cells with Pax7 (green), MyoD1 (red), and DAPI (blue). Scale bar 100 μm. (B) Rate of expressed Pax7 and Myod1 in total nuclei (DAPI) at satellite cell and fibroblast cell. n = 3, The asterisks represent significant differences in expression levels (Student’s *t*-test): * p<0.05; ** p<0.01; *** p<0.001; **** p<0.0001. Error bars show±standard deviation. (C) Morphology of satellite cell from various ECMs at early passage (P2) and late passage (P7). Scale bar 100 μm. (D) Doubling time of satellite cell in various ECM at early passage (P2) and late passage (P7). (E) The cell cycle of the satellite cell was stained with propidium iodide (PI) and analyzed with flow cytometry. ECM, extra-cellular matrix; Pax7, paired box 7; Myod1, myogenic differentiation 1; DAPI, 4′6-diamidino-2-phenylindole.

Porcine muscles were isolated into satellite cells and fibroblasts using pre-plating methods (Figure 1A). A previous report showed that Pax7 is a core regulation factor of satellite cells and Myod1 is an implicated myogenic regulator factor [20,21]. The number of Pax7 and MyoD1 positive cells in the isolated and cultured cells was measured by ICC. Levels of expressed Pax7 were higher in the satellite cell than in the fibroblast (p<0.0001) (Figure 1B) (satellite cell: 72% [±0.21], fibroblast cell 9.1% [±0.02]). Porcine satellite cells expressed *MYOD1* gene at 64% (±0.16) in total cultured cell while fibroblast cells expressed *MYOD1* gene at 10% (±0.02) in total cultured cell (p<0.001) (Figure 1B). This result indicates that porcine satellite cells were effectively isolated from muscle tissues by pre-plating.

To determine proliferation capability of 0.1% gelatin-, fibronectin (20 μg/mL)- and non-coated dishes, we measured cell doubling time. The doubling time in the porcine satellite cells was measured at early passage (P2) and late passage (P7) in each of the ECM. The doubling time of gelatin-coated satellite cells had increased from 30.2 (±0.33 h) to 55.3 (±0.68 h) between the early and late passage. The time for the non-coated satellite cells increased from 85 h (±18.6) to 204.4 h (±36.6) between the early and late passage (Figure 1D). Interestingly, the doubling time of 20 μg/mL of fibronectin-coated satellite cells at the early passage and late passage had slightly increased from 31.3 h (±3.1) to 35.4 h (±2.1) (Figure 1C–1D). This result indicates that fibronectin-coated satellite cells could support proliferation during the time elapsed between the early and late passage.

We next analyzed the cell cycles of the porcine satellite cells from different ECMs using measurements taken in an early and late passage. All S phases were decreased in each of the three types of coated dishes during late passage (P2, gelatin 18.9% [±0.04]; fibronectin, 18.87% [±0.02]; non-coated 22.81% [±0.22]; P7, gelatin 17.45% [±0.89]; fibronectin 15.26% [±0.94]; non-coated 17.73% [±0.92]) (Figure 1E). However, the G0/G1 phase had increased by the time of the late passage (P2, gelatin 58.9% [±1.05], fibronectin, 62.55% [±0.29]; non-coated 55.65% [±0.23]; P7, gelatin 72.31% [±1.5]; fibronectin 74.46% [±1.14]; non-coated 70.26% [±1.27]) (Figure 1E). Our result indicates that S phase between different ECM had no significant differences, and the S phase was consistent in decreasing across all different ECM types.

### Porcine satellite cells on fibronectin-coated dishes were maintained *in vitro* at the time of the early and late passages

**Figure 2:**
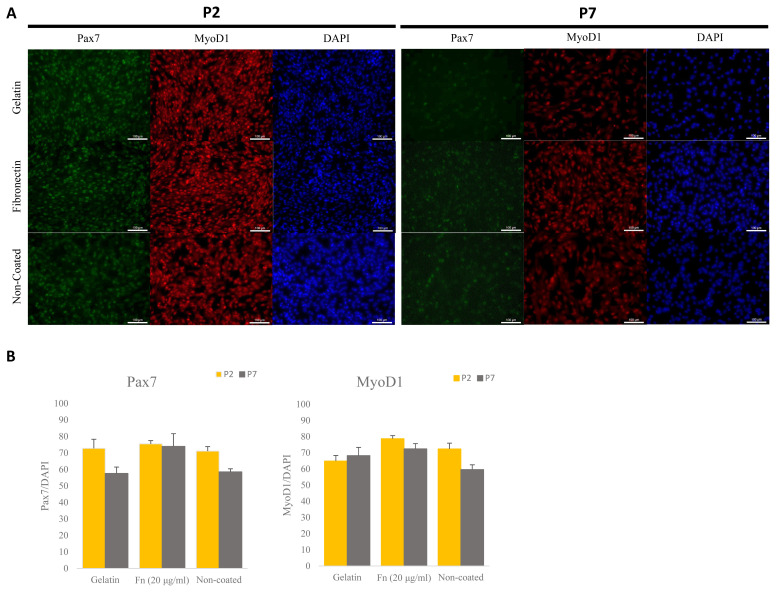
Comparative analyses of expressed Pax7 and Myod1 via immunocytochemistry (ICC) at different ECMs at early and late passage. (A) Immunostaining of Pax7 (green), MyoD1 (red), and DAPI (blue) at early and late passage. Scale bar 100 μm. (B) Rate of expressed Pax7+/DAPI and Myod1+/DAPI in porcine satellite cells at early and late passage with gelatin-, fn (20 μg/mL)- or non-coated dishes. Pax7, paired box 7; Myod1, myogenic differentiation 1; ECMs, extra-cellular matrix; DAPI, 4′6-diamidino-2-phenylindole.

To determine the effect of maintenance of porcine satellite cells cultured on different ECM *in vitro*, we analyzed expression levels of the *PAX7* and *MYOD1* genes via ICC (Figure 2A). Porcine satellite cells cultured on fibronectin (20 μg/mL) expressed higher levels of *PAX7* genes than porcine satellite cells cultured on gelatin or non-coated dishes in early and late passage (Figure 2B) (P2, gelatin 72.80% [±5.47]; fibronectin, 75.51% [±1.98]; non-coated 71.12% [±2.71]; P7, gelatin 57.56% [±3.89]; fibronectin 73.97% [±7.75]; non-coated 58.52% [±1.87]). Also porcine satellite cells cultured on fibronectin (20 μg/mL) expressed high levels of MyoD1 at both the time of the early and late passage (Figure 2B) (P2, gelatin 64.89% [±3.46]; fibronectin, 78.72% [±1.94]; non-coated 72.38% [±3.57]; P7, gelatin 68.27% [±5.10]; fibronectin 72.44% [±3.26]; non-coated 59.62% [±2.99]). These results indicate that porcine satellite cells are efficiently maintained on fibronectin.

### Optimizing fibronectin concentration for proliferation of porcine satellite cells

**Figure 3:**
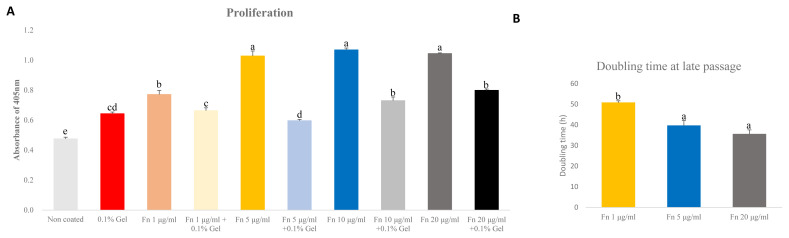
Comparative analyses of proliferation of satellite cell on various concentration of fibronectin (Fn) and gelatin (Gel) mixture at early passage. Comparative analysis of doubling time of various concentration of fibronectin at late passage. (A) Cell proliferation was analyzed by cell counting kit-8 (CCK-8). Analysis was undergone after treating CCK-8 for 3 hours. n = 7, each letter ^a–d^ represents significant differences (p<0.0001). (B) Cell doubling time was analyzed with a doubling time formula. Each letter ^a,b^ represents significant differences (p<0.05).

The ideal concentration of fibronectin as applied to porcine satellite cells to encourage proliferation is unclear. We analyzed the proliferation using different concentrations of fibronectin measured in an early passage. Moreover, as the mixture of gelatin and fibronectin exerted a positive effect on chondrogenesis, we analyzed the proliferation of mixed gelatin and fibronectin at various concentrations [19]. Fibronectin (5, 10, 20 μg/mL) was the most effective (p<0.0001) at supporting proliferation (Figure 3A). Interestingly, all concentrations of fibronectin/gelatin mixture resulted in less proliferation than a single coating of fibronectin. As there were no differences in proliferation observed between different concentrations of fibronectin, and 1 μg/mL of fibronectin increased proliferation more than gelatin, we narrowed the tested fibronectin concentrations to 1 μg/mL, 5 μg/mL and compared these with 20 μg/mL of fibronectin. We cultured these concentrations to late passage and analyzed the doubling time of the porcine satellite cells. The 5 μg/mL and 20 μg/mL of fibronectin showed the lowest doubling time, indicating that cell proliferation was effective at 5 μg/mL and 20 μg/mL (p<0.05) (Figure 3B).

### Analysis of cell cycle in satellite cells grown with different concentrations of fibronectin

**Figure 4:**
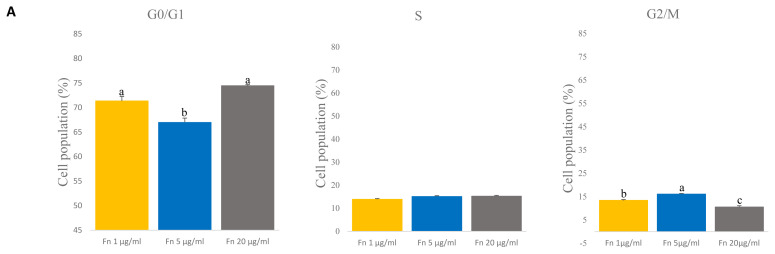
Comparative analyses of cell cycle on various concentration of fibronectin (fn) at time of late passage. (A) The cell cycle of the satellite cell was stained with propidium iodide (PI) and analyzed with flow cytometry. G0/G1 phase: n = 3, each letter ^a,b^ represents significant differences (p<0.005). G2/M phase: n = 3, each letter ^a–c^ represents significant differences (p<0.005).

To determine the cell cycle of satellite cells grown with different concentrations of fibronectin, we used a flow cytometer. Fibronectin 1 and 20 μg/mL showed the highest percent of G0/G1 phase than 5 μg/mL (p<0.005) (Figure 4A). However, the G2/M phase was significantly higher with the 5 μg/mL (p<0.005) (Figure 4A). No significant differences in the S phase were observed across concentrations (Figure 4A). This result indicates that there are no differences in the S phase when different concentrations of fibronectin are used.

### Optimizing fibronectin concentration for maintenance of porcine satellite cells

**Figure 5:**
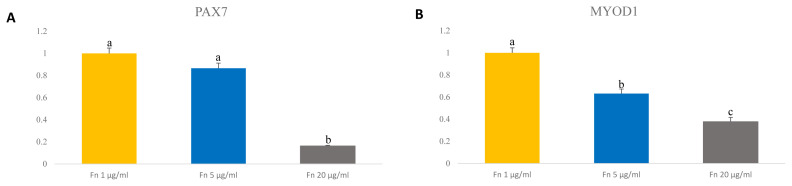
Expression level of transcription factor *PAX7* and *MYOD1* in porcine satellite cells at late passage. (A) Level of gene expression of *PAX7* in porcine satellite cells. n = 3, Each letter ^a,b^ represents significant differences (p<0.0001). (B) Level of gene expression of MyoD1 in porcine satellite cells. n = 3, Each letter ^a–c^ represents significant differences (p<0.0001). *PAX7*, paired box 7; *MYOD1*, myogenic differentiation 1.

Previous reports showed that Pax7 expression levels decrease rapidly as cells are cultured over a long period [13,22]. We analyzed expression level of Pax7 and myogenic transcription factor MyoD1 by qRT-PCR to determine the expression levels at different concentrations of fibronectin. The results showed that *PAX7* gene expression levels were highest with fibronectin 1 μg/mL and with fibronectin 5 μg/mL and lowest in 20 μg/mL fibronectin (p<0.0001) (Figure 5A). Interestingly, the expression level on *MYOD1* gene were down regulated in concentration of 5 μg/mL and 20 μg/mL compared to 1 μg/mL (p<0.0001) (Figure 5B). Surprisingly, fibronectin 20 μg/mL showed the lowest expression levels of Pax7 and MyoD1. This result suggests that even a low concentration of fibronectin maintains Pax7 expression and can potentially maintain the satellite cell as well.

## DISCUSSION

Many researchers have attempted to maintain satellite cells *in vitro* by regulating the associated signal pathway, growth factors, and ECM. In 2009, studies on porcine muscle stem cell characteristics for various ECMs had been conducted [14]. However, to date there has been no research covering how the characteristics of long term cultured porcine satellite cells depend on the ECM used. Fibronectin is one ECM already widely used in cell cultures. Here we analyzed the characteristics of short- and long-term culture of porcine satellite cells cultured on 20 μg/mL of fibronectin-, gelatin- and non-coated dishes. Our result showed that 20 μg/mL of fibronectin could proliferate and maintain the cells better than gelatin-coated and non-coated dishes. We also found that 5 μg/mL of fibronectin had was the optimal level for significant proliferation and maintenance of porcine satellite cells.

Fibronectin cooperates with growth factors to induce cell signal pathway. The FGFs are essential in satellite cells which regulate cellular self-renewal and differentiation. In mouse satellite cells, fibronectin and β1-integrin cooperate with FGF and enhance phosphorylation of Erk, an AKT signal pathway that regulates cell proliferation [23]. In satellite cells, fibronectin stimulates wnt7a through the FZD7/Sdc4 coreceptor complex. Fibronectin and Wnt7a together regulate the population level of satellite cells and satellite myogenic cells [24]. Also, it has been reported that fibronectin-integrin signaling regulates the Hippo pathway via the FAK-Src-PI3K-3-phosphoinositide dependent protein kinase 1 (*PDK1*) gene [25,26]. In short, there is support for our finding that proliferation and expression of Pax7 and Myod1 increases in fibronectin-coated dishes.

It is found that the capability of cell adhesion to culture dish can lead to cell proliferation. Previous reports have shown that endothelial cells placed onto polytetrafluorchentlyne vascular grafts could be effectively attached with fibronectin. The 2 μg/mL of fibronectin had the lowest attachment rate when compared to 10, 20, 50, 100 μg/mL of the same. Also, when 10 μg/mL fibronectin was used, there was a difference in the attachment rate until the 10minute mark, however, as time progressed, no significant difference between this sample and those with fibronectin at higher concentrations emerged. It has also previously been found that fibronectin 20, 50, 100 μg/mL show no statistical differences in their attachment rate between 0 to 120 minutes [27]. In our doubling time results, we observed a high doubling time when 1 μg/mL of fibronectin was used, more so than with 5 μg/mL and 20 μg/mL. Also, no differences in 5 μg/mL and 20 μg/mL were observed, which is consistent with the endothelia cell attachment results. Ultimately, we conclude that in satellite cells there is no difference in doubling time above 5 μg/mL, but doubling time is lower with 1 μg/mL. There were also no differences in the S phases of the samples using different concentrations of fibronectin. This leads us to conclude that the 5 μg/mL concentration of fibronectin is optimal for porcine satellite cell adherence and proliferation.

In the *in vivo* niche, many ECMs interact with one another to maintain the niche. To mimic the microenvironment *in vitro* many studies have been conducted mixing the ECMs with various cell types. Binding between collagen and fibronectin could cause conformation changes in fibronectin and could result in a biological response such as cell attachment, migration, and wound repair [28]. In a C2C12 myoblast, a fibronectin-collagen cell showed a significant increase in differentiation [29]. A mixture of gelatin and fibronectin provided during chondrogenesis was shown to enhance chrodrogeinc genes such as SRY-box transcription factor 9 (*SOX9*) and collagen type 2 alpha 1 chain (*COL2A1*) and could improve the niche of chondrogenesis [19]. However, gelatin and fibronectin mixed at various ratios and provided to porcine satellite cells had no significant effect of proliferation (Figure 3A).

Previous studies have shown that the characteristics of cells change depending on the fibronectin concentration. In MCF-7 cells, a Zymographic analysis result showed that a prominent matrix metalloproteinase-2 (MMP-2) and MMP-9 band appeared when exposed to a concentration of 20 μg/mL. At concentrations of 5 μg/mL and 10 μg/mL MMP-2 was barely detectable and MMP intensity was much less [30]. In a satellite cell culture, expression levels of Pax7 must be maintained, as this lowers throughout the long-term culture [13,22]. Our results, achieved using porcine satellite cells, showed that *PAX7* gene expression levels were high in the presence of a low concentration of fibronectin. Additional studies will be needed to further determine the relationship between the maintenance of porcine satellite cells and the concentration.

## CONCLUSION

We examined the effect of porcine satellite cells on fibronectin during a long-term culture and identified the most appropriate concentration of fibronectin necessary to maintain the satellite cells. Experimentation showed that concentrations of 5 μg/mL maintained Pax7 expression up to the time of a late passage and resulted in a low doubling time, suggesting their potential as a means of maintaining porcine satellite cells. We also showed that the characteristics of the satellite cells could change depending on the ECM used and its concentrations. This study provides some essential information concerning the effective culturing of porcine satellite cells and the criteria that should be applied as an ECM is decided upon.
